# Effects of temperature fluctuations and phase transitions on quality attributes of frozen Korean white kimchi

**DOI:** 10.1016/j.fochx.2025.102610

**Published:** 2025-06-03

**Authors:** Miran Kang, Eunji Kim, Hyun-Jung Chung, Sung Hee Park

**Affiliations:** a Kimchi Industry Promotion Division*,* World Institute of Kimchi*,* Gwangju *61755,* Republic of Korea; b Division of Food and Nutrition*,* Chonnam National University*,* Gwangju*, 61186,* Republic of Korea; c Fusion Material Research Group*,* Korea Institute of Materials Convergence Technology*,* Busan *47154,* Republic of Korea

**Keywords:** Glassy state, Frozen storage, Korean white kimchi, Principal component analysis, Phase transition

## Abstract

With global demand for kimchi rising, effective preservation methods that maintain quality and extend shelf life are increasingly important. This study evaluated the effects of temperature fluctuations and phase transitions on the microstructure and physicochemical properties of Korean white kimchi during 4 weeks of frozen storage under four conditions: glassy state at −45 °C without fluctuations, and glassy, rubbery, and partially thawed states with fluctuations. Although pH, titratable acidity, and reducing sugars remained stable, ice crystal formation, microstructure, drip loss, hardness, colour, and antioxidant activity were significantly affected. Principal component analysis revealed that storage time and phase transitions influenced the distribution of quality variables, resulting in clearer sample separation. Phase transition conditions thus affected quality changes during frozen storage. Storage in the glassy state without fluctuations suppressed large ice crystal formation and preserved structure and function. These findings support glassy-state storage as a practical strategy for long-term kimchi preservation.

## Highlights

• Phase transitions and temperature fluctuations significantly affected the quality of frozen KWK.
• Four storage conditions were tested: glassy (with or without fluctuations), rubbery, and partially thawed.
• Glassy-state storage without fluctuations effectively suppressed structural damage and quality loss.
• Glassy-state storage is proposed as a practical strategy for long-term kimchi preservation.

## Nomenclature

DSC: differential scanning calorimeter
KWK: Korean white kimchi
LAB: lactic acid bacteria
PCA: principal component analysis
RSC: reducing sugar content
TA: titratable acidity
*T*_g_*'*: glass transition temperature
*T*_m_*'*: melting temperature

## 1 Introduction

Kimchi, a traditional Korean fermented vegetable dish, is rich in functional ingredients and has gained significant global attention, particularly after the COVID-19 pandemic, because of its numerous health benefits, including anti-obesity, anti-inflammatory, anti-tumour, antioxidant, and immunomodulatory effects (Yun et al., 2022). There are approximately 300 varieties of kimchi, classified based on ingredients, seasonings, and preparation methods (Sin & Cho, 2017). Among these, Korean white kimchi (KWK) stands out for its mild, non-spicy flavor and pale appearance, as does not contain red pepper powder (Kim et al., 2021). Typically made with kimchi cabbage and water-based seasonings such as garlic, ginger, and pear, KWK is popular among children and those seeking a milder, lower-sodium option (Sin & Cho, 2017). At the same time, growing international demand for kimchi, particularly in the United States and Europe, has led to an increase in its exportation (Park, Kim, Lee, et al., 2024). However, the lactic acid fermentation process causes kimchi to reach its optimal ripeness (pH 4.2–4.5) within 2 weeks under standard refrigeration (4–10 °C), resulting in carbon dioxide accumulation from hetero-type lactic acid bacteria (LAB) (Baek et al., 2020). This accumulation can lead to swollen packaging, complicating long-term storage and distribution, particularly during sea transport, which typically takes over 20 days to complete (Park et al., 2022; Park, Kim, Lee, et al., 2024). Therefore, techniques such as heat treatment (Lee et al., 2019), irradiation (Park et al., 2023), and the use of antimicrobials (Kang et al., 2019), chemical additives (Ko et al., 2012), and microbial additives (Jang et al., 2015) have been employed to preserve kimchi. However, although these methods can slow the fermentation process, they have limitations in effectively maintaining product quality during storage and distribution for commercial use.

Freezing is widely used to extend the shelf life of high-moisture foods by inhibiting microbial growth and slowing chemical and enzymatic reactions (Kang et al., 2019; Kumar et al., 2019). The effectiveness of freezing for preserving food quality is highly dependent on the storage temperature used, with the glass transition temperature (*T*_g_*'*) playing a critical role. Storing foods below the *T*_g_*'* minimises molecular mobility, preventing quality deterioration and maintaining stability in the glassy state (Xu et al., 2020). This strategy has proven effective in preserving the quality of various foods, including celery (Xu et al., 2020), potatoes (Kumar et al., 2019), and mangoes (Zhang et al., 2017). Recent research also indicates that rapid freezing and storage at −45 °C (below *T*_g_*'*) can effectively preserve the physicochemical quality of frozen kimchi while minimising histological damage (Kang et al., 2024). Furthermore, storing kimchi at temperatures below −40 °C has been reported to be effective in preserving the LAB count after thawing (Park, Kim, Lee, et al., 2024).

The *T*_g_*'* is particularly important as it marks the point where food transitions from a rigid, glassy state to a more flexible, rubbery state. Beyond this point, the melting temperature (*T*_m_*'*) becomes critical, where ice crystals begin to melt, leading to increased water mobility and potential recrystallisation (Lim et al., 2006). These phase transitions can be detrimental, especially during temperature fluctuations throughout storage and distribution, leading to structural damage through ice crystal growth and changes in the microstructure and physicochemical properties of food (Xu et al., 2020). Maintaining storage temperatures below the *T*_g_*'* is, therefore, crucial to prevent such degradation, as exceeding the *T*_g_*'* or *T*_m_*'* can significantly impact the texture, colour, and nutritional content of food (Kumar et al., 2020).

Despite the widespread use of freezing for food preservation, there is a notable gap in research specifically investigating the effects of phase transitions caused by temperature fluctuations on the quality of frozen kimchi. Understanding how various phase transition conditions influence the quality of frozen kimchi is critical for overcoming current limitations in its long-distance distribution and export. This research is especially relevant in the context of global commercialisation, where maintaining product quality during extended frozen storage is essential for market competitiveness.

This study aimed to investigate the impact that these transitions and temperature fluctuations over 4 weeks of storage have on the physicochemical properties, ice crystal formation, microstructure, drip loss, hardness, colour, and antioxidant activity of kimchi. Principal component analysis (PCA) was applied to evaluate and visualise the multivariate quality changes of frozen KWK under different phase transition conditions. The results are expected to contribute to the optimisation of storage conditions for maintaining the quality of kimchi during extended frozen storage.

## 2 Materials and methods

### 2.1 Sample preparation

Unripe KWK samples were collected from a kimchi production facility in Gwangju, Republic of Korea, on the day of the experiment. The composition of KWK per 100 g included 60.40 g kimchi cabbage; 22.70 g purified water; 4.80 g ground pear; 1.50 g salt; 1.20 g each of paprika, ground radish, ground onion, sliced carrot, and ground garlic; 1.00 g each of fermented shrimp and anchovy juice; 0.80 g each of ground ginger and glutinous rice paste; and 0.50 g sugar. The moisture content and salinity of unripe KWK immediately after purchase were 93.83 ± 0.24 % and 0.80 ± 0.05 %, respectively. For subsequent frozen storage experiments, the midrib portions of the cabbage were cut into 3 × 3 cm pieces.

### 2.2 Determining storage conditions via phase transition characteristics

**Fig. 1:**
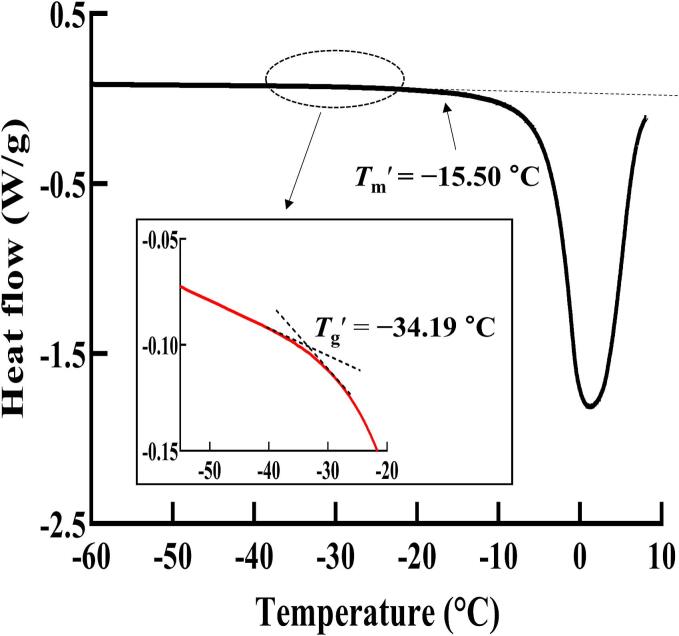
Determination of the glass transition temperature (*T*_g_*'*) and melting temperature (*T*_m_*'*) of Korean white kimchi using differential scanning calorimetry analysis.

The phase transitions of unripe KWK were analysed using a differential scanning calorimeter (DSC 4000; PerkinElmer Inc., Waltham, MA, USA) in a nitrogen atmosphere with a flow rate of 19.8 mL/min (Syamaladevi et al., 2012; Tian et al., 2020). KWK samples weighing 20 mg were placed in stainless-steel pans, with an empty pan serving as the reference. All pans were sealed and stabilised at 4 °C for 30 min. During DSC analysis, the temperature was initially decreased from 4 °C to −70 °C at a rate of 10 °C/min, held for 2 min, and then increased back to 4 °C at a rate of 5 °C/min. The *T*_g_*'* was identified from the peak on the first-derivative curve of the DSC thermogram, whereas the *T*_m_*'* was determined at the intersection between the baseline and the left side of the endothermic peak. The *T*_g_*'* and *T*_m_*'* of KWK were identified to be −34.19 °C and −15.50 °C, respectively (Fig. 1), based on heat flow curve changes in the DSC thermogram. Phase transition temperature settings were determined with reference to Zhang et al. (2017). KWK stored at −45 °C in a glassy state without temperature fluctuations was designated as *T*_1_. Additional groups included KWK samples exposed to −40 °C, −25 °C, and −10 °C, corresponding to glassy (*T*_2_ < *T*_g_*'*), rubbery (*T*_g_*'* < *T*_3_ < *T*_m_*'*), and partially thawed (*T*_4_ > *T*_m_*'*) states, respectively. Although −45 °C and −40 °C are below *T*_g_*'* (−34.19 °C), they were distinguished to evaluate the effects of temperature fluctuations within the glassy state.

### 2.3 Sample packaging and phase-transition conditions

Each 200 g KWK sample was vacuum-packaged in a low-density polyethylene film bag (17.7 × 18.8 cm; Thai Griptech Co., Ltd., Bangkok, Thailand) using a vacuum sealer (Airzero AZC-070; INTRISE, Ansan, Republic of Korea). Samples were frozen using an ultralow temperature immersion freezer (F-500; Topgreentech, Seoul, Republic of Korea) equipped with a jet stirrer (Kang et al., 2024). Temperature fluctuation treatments were applied twice daily to induce specific phase transition states, as defined in Section 2.2. The *T*_1_ group was stored at −45 °C without temperature fluctuations. The *T*_2,_ *T*_3_, and *T*_4_ groups were exposed to room temperature (25 °C) for durations required to reach target temperatures of −40, −25, and −10 °C, requiring 1, 8, and 21 min, respectively. These times were measured using a fibre optic temperature sensor (FOTEMP4; Optocon AG, Dresden, Germany). For weekly analyses, frozen KWK samples were thawed in a 25 °C incubator. Thawing was continuously monitored in real time using the fibre optic temperature sensor and was terminated once the core temperature reached 0 °C. As commercially available KWK is typically consumed directly after thawing, no further processing, such as washing, draining, or cutting, was performed after freezing and thawing.

### 2.4 Ice crystal morphology and microstructure

The ice crystal morphology and microstructure of frozen KWK samples were examined according to the method described by Kang et al. (2024). Frozen KWK samples were sliced into pieces (5 × 10 × 1 mm) while kept on ice. Images of the ice crystal structure were captured in a laboratory maintained at −10 °C using a fluorescence microscope (BX53; Olympus, Tokyo, Japan) equipped with a digital camera (DP28; Olympus) at a 200× magnification. Ice crystal quantity, diameter, and surface area were measured using the Digimizer image analysis software (version 6.4.0; MedCalc Software, Ostend, Belgium).

For microstructure observation, KWK samples were freeze-dried and cut into 2 × 2 × 1 mm blocks. The samples were then coated with platinum using a sputtering device (JFC-1500; JEOL, Tokyo, Japan). High-resolution images of the prepared samples were obtained using a field-emission scanning electron microscope (Gemini500; Carl Zeiss AG, Oberkochen, Germany) at a 50× magnification with an accelerating voltage of 15 kV.

### 2.5 Drip loss

Frozen 200 g KWK samples were thawed at weekly intervals to calculate drip loss using Eq. (1) below:

(1) $$\text{Drip loss}\;\left(\%\right)=\left(W_{1}-W_{2}\right)/W_{1}\times 100$$

where *W*_1_ and *W*_2_ represent the weight (g) of the KWK samples before and after thawing, respectively. Measurements were performed for five replicates.

### 2.6 pH, titratable acidity (TA), and reducing sugar content (RSC)

To measure the pH and TA of the KWK samples, 100 g of kimchi was homogenised for 1 min using a blender (HR2535; Philips, Amsterdam, The Netherlands) and filtered through sterile gauze. The pH of the filtrate was measured at 25 °C using a pH meter (Orion Versa Star Pro; Thermo Scientific, Waltham, MA, USA). TA was determined in accordance with AOAC method 942.15 (AOAC International, 2023) by titrating 10 mL of the filtrate with 0.1 N NaOH until the pH reached 8.3. The TA was calculated as the percentage of lactic acid using Eq. (2) below:

(2) $$\mathrm{TA}\;\left(\%\text{lactic acid}\right)=\left(V\times F\times 0.009\right)/W\times 100$$

where *V* is the volume of 0.1 N NaOH used (mL), *F* is the factor of NaOH, and *W* is the sample weight (g).

For the analysis of RSC, the homogenised sample was diluted 50-fold with distilled water and filtered using Whatman No. 2 filter paper. A 1 mL aliquot of the filtrate was mixed with 3 mL of 3,5-dinitrosalicylic acid reagent (Sigma-Aldrich, St. Louis, MO, USA), heated at 100 °C for 5 min, and cooled for 10 min. The absorbance of the resulting solution was measured at 550 nm using a microplate reader (SPECTRO Star Nano; BMG Labtech, Ortenberg, Germany). RSC (mg/g) was calculated based on a standard glucose curve using Eq. (3) below:

(3) $$\mathrm{RSC}\;\left(\mathrm{mg}/g\right)=\left(C\times D\right)/W$$

where *C* is the glucose-equivalent concentration from the standard curve (mg/g), *D* is the dilution factor, and *W* is the sample weight (g).

### 2.7 Instrumental hardness and colour

The hardness of thawed KWK samples was evaluated at room temperature using a texture analyser (TA-XT2; Stable Micro Systems Ltd., Godalming, UK) fitted with a cylindrical probe (diameter = 2 mm). The central portion of KWK flesh was penetrated with the probe at a speed of 0.5 mm/s. The hardness values, expressed in Newtons (N), were obtained by recording the peak force at the fracture point.

Instrumental colour parameters, including CIE *L*^⁎^ (lightness), *a*^⁎^ (redness/greenness), and *b*^⁎^ (yellowness/blueness), of the KWK sample were measured using a colourimeter (CR-400; Konica Minolta, Tokyo, Japan) calibrated with a standard calibration plate. The overall colour variation among the samples (Δ*E*) was computed using Eq. (4) below:

(4) $$\Delta E^{*}={\left[{\left(L^{*}-{L_{0}}^{*}\right)}^{2}+{\left(a^{*}-{a_{0}}^{*}\right)}^{2}+{\left(b^{*}-{b_{0}}^{*}\right)}^{2}\right]}^{1/2}$$

where *L*_0_^⁎^, *a*_0_^⁎^, and *b*_0_^⁎^ represent the colour parameters of the reference sample, whereas *L*^⁎^, *a*^⁎^, and *b*^⁎^ are those of the tested sample. A 0-day frozen KWK sample was used as a reference.

### 2.8 Antioxidant activity

The DPPH free radical-scavenging activity of the samples was measured according to the method described by Park, Kim, Park, et al. (2024), with minor modifications. Briefly, 1 mL of the kimchi methanol extract and 10 mL DPPH solution were mixed (1:10, *v*/v) and placed in the dark for 1 h. The absorbance was then measured at 517 nm with a microplate reader. The percentage of DPPH inhibited in the sample was calculated using Eq. (5) below:

(5) $$\text{DPPH radical}-\text{scavenging activity}\;\left(\%\right)=\left[\left(A_{\text{control}}-A_{\text{sample}}\right)/A_{\text{control}}\right]\times 100$$

### 2.9 Statistical analysis

The experimental results are presented as the mean ± standard deviation from three independent replicates. Statistical analysis was performed using JMP version 18 (SAS Institute Inc., Cary, NC, USA). One-way ANOVA was conducted, followed by Tukey's HSD test for post-hoc comparisons. Statistical significance was set at a *p*-value <0.05. Additionally, the effects that storage period and temperature fluctuations have on the quality attributes of frozen samples, as well as their interactions, were analysed using two-way ANOVA. PCA was also performed using the same software to visualise multivariate patterns and sample clustering based on quality attributes during frozen storage. Prior to PCA, all variables were standardised using *Z*-score transformation (i.e., mean-centred and scaled to unit variance) to ensure comparability. Score and loading plots were generated to illustrate sample distribution and the contribution of each variable to the principal components.

## 3 Results and discussion

### 3.1 Ice crystal structure and microstructure

**Fig. 2:**
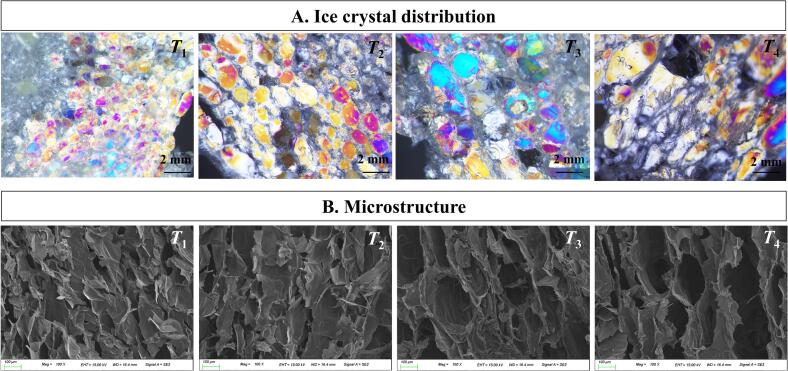
Ice crystal distribution (A) and microstructure (B) of frozen Korean white kimchi after four weeks of storage with or without phase transitions. Scale bars = 2 mm. *T*_1_, glassy state without temperature fluctuations (−45 °C); *T*_2_, glassy state (*T* < *T*_g_*'*); *T*_3_, rubbery state (*T*_g_*'* < *T* < *T*_m_*'*); *T*_4_, partially thawed state (*T* > *T*_m_*'*). *T*_2_–*T*_4_ were stored under fluctuating temperature conditions.

The size and distribution of ice crystals affect microstructure integrity, which are crucial factors that influence the quality of frozen food (Neri et al., 2014). Polarised images of the ice crystals that formed in frozen KWK samples after 4 weeks of storage with or without phase transitions are presented in Fig. 2A. *T*_1_ and *T*_2_ showed small, uniformly distributed ice crystals. Conditions with temperatures below the *T*_g_*'*, and especially without temperature fluctuations, prevented the clustering and growth of ice crystals. Similar results were reported by Syamaladevi et al. (2012), indicating that reduced molecular mobility at low temperatures limits recrystallisation. In contrast, *T*_3_ and *T*_4_ exhibited irregular and larger ice crystals as a result of recrystallisation. This aligns with the understanding that temperature fluctuations promote the melting and refreezing of ice, leading to the growth of larger ice crystals (Vicent et al., 2020).

The primary attributes used to assess ice crystal size are diameter and area (Dalvi-Isfahan et al., 2019). Temperature fluctuations decreased the number of ice crystals while increasing their size (Table 1). The number of ice crystals in frozen storage without temperature fluctuations (*T*_1_) was the highest at 73.80, whereas the counts in frozen storage with temperature fluctuations (*T*_2_–*T*_4_) ranged from 14.40 to 40.60, indicating that *T*_1_ had significantly higher values compared with those of the other conditions (*p* < 0.05). The diameters and areas of ice crystals in the glassy and rubbery states (*T*_1_–*T*_3_) ranged from 0.77–1.38 mm and 0.33–3.39 mm^2^, respectively. In contrast, the partially thawed state (*T*_4_) exhibited significantly larger ice crystals, with a mean diameter of 3.88 mm and an area of 10.86 mm^2^ (*p* < 0.05). Consistent with our findings, previous studies showed that temperature fluctuations during frozen storage increased ice crystal size and decreased the number of ice crystals in frozen potato and salmon tissues (Syamaladevi et al., 2012; Ullah et al., 2014).

**Table 1:** Changes in the number, diameter, and area of ice crystals in frozen Korean white kimchi after 4 weeks of storage with or without phase transitions.

Temperature fluctuation	Ice crystal parameter		
	Number	Diameter (mm)	Area (mm^2^)
*T*_1_	73.80 ± 4.09^A^	0.77 ± 0.10^B^	0.33 ± 0.05^C^
*T*_2_	40.60 ± 3.51^B^	1.09 ± 0.19^B^	1.15 ± 0.36^BC^
*T*_3_	24.80 ± 2.39^C^	1.38 ± 0.50^B^	3.39 ± 0.97^B^
*T*_4_	14.40 ± 2.70^D^	3.88 ± 1.37^A^	10.86 ± 3.06^A^

All data are presented as the mean ± standard deviation. Different letters (A–D) within the same column indicate significant differences (*p* < 0.05). *T*_1_, glassy state without fluctuations (−45 °C); *T*_2_, glassy state (*T* < *T*_g_*'*); *T*_3_, rubbery state (*T*_g_*'* < *T* < *T*_m_*'*); *T*_4_, partially thawed state (*T* > *T*_m_*'*). Ice crystal number, diameter, and area were determined with a total of *n* = 9 (three replicates, each with three different photographs).

The analysis of the microstructure of frozen KWK samples stored under various temperature fluctuation conditions for 4 weeks is shown in Fig. 2B. In *T*_1_, a dense structure with small pores was observed, indicating minimal damage under stable glassy-state conditions without temperature fluctuations. In *T*_2_, although temperature fluctuations were applied, the structure remained relatively intact due to the glassy state being maintained below *T*_g_*'*, which limited molecular mobility. In contrast, *T*_3_ and *T*_4_, stored in rubbery and partially thawed states, showed more severe structural damage caused by increased molecular mobility and water migration. Temperature fluctuations induce recrystallisation, leading to the formation of irregular and large ice crystals, impacting the optimal microstructure and quality of frozen vegetables (Vicent et al., 2020). Similarly, in frozen mangoes, phase transitions resulted in larger ice crystals and an irregular microstructure (Zhang et al., 2018). These results suggest that storing frozen KWK at −45 °C, a temperature below the *T*_g_*'*, without temperature fluctuations, effectively preserves the cellular structure by suppressing the formation and recrystallisation of large ice crystals.

### 3.2 Drip loss

In frozen plant-based foods, drip loss is a crucial marker directly linked to weight loss, sensory attributes, water retention, and various physicochemical properties. (Li et al., 2023; Xu et al., 2020). It increases as a result of cell wall destruction from the resizing and redistribution of ice during frozen storage, accompanied by nutrient leakage (Vicent et al., 2018). The changes in drip loss of frozen KWK samples during 4 weeks of storage with or without phase transitions are presented in Table 2. The drip loss of KWK was 23.83 % immediately after freezing. After 4 weeks of storage, the drip loss of KWK in the glassy state (*T*_1_ and *T*_2_) increased to 28.08–37.59 %, which was significantly lower than the increases observed in the rubbery (*T*_3_) and partially thawed (*T*_4_) states (*p* < 0.05), which were 44.85 % and 51.82 %, respectively. Two-way ANOVA confirmed that the storage period, temperature fluctuations, and their interaction had a significant impact on the drip loss of KWK (*p* < 0.001). Ice crystal growth and microstructural changes resulting from temperature fluctuations, as shown in Fig. 2, support the increase in drip loss observed. This aligns with previous studies on frozen mangoes, where the lower drip loss measured in the glassy state compared with that in the rubbery state was attributed to a controlled diffusion-limited reaction (Zhang et al., 2017). Therefore, storage in the glassy state without temperature fluctuations minimises drip loss and preserves the quality of frozen KWK samples for a longer period.

**Table 2:** Changes in the drip loss of frozen Korean white kimchi during 4 weeks of storage with or without phase transitions.

Parameter	Temperature fluctuation	Storage period (week)					Levels of significance		
		0	1	2	3	4	SP	TF	SP × TF
Drip loss (%)	*T*_1_	23.83 ± 1.35^Ab^	25.24 ± 1.64^Cab^	26.90 ± 1.45^Bab^	27.57 ± 1.79^Bab^	28.08 ± 0.60^Da^	***	***	***
	*T*_2_	23.83 ± 1.35^Ad^	27.26 ± 1.10^Ccd^	31.33 ± 2.89^Bbc^	34.50 ± 1.80^Bab^	37.59 ± 0.47^Ca^			
	*T*_3_	23.83 ± 1.35^Ad^	33.68 ± 1.27^Bc^	39.19 ± 2.96^Abc^	42.85 ± 2.64^Aab^	44.85 ± 1.53^Ba^			
	*T*_4_	23.83 ± 1.35^Ad^	38.45 ± 2.45^Ac^	42.18 ± 3.22^Abc^	48.31 ± 5.02^Aab^	51.82 ± 3.76^Aa^			

SP, storage period; TF, temperature fluctuation.

All data are presented as the mean ± standard deviation. Different uppercase letters (A–D) within the same column and different lowercase letters (a–d) within the same row indicate significant differences (*p* <0.05). *T*_1_, glassy state without fluctuations (−45 °C); *T*_2_, glassy state (*T* < *T*_g_*'*); *T*_3_, rubbery state (*T*_g_*'*< *T* < *T*_m_*'*); *T*_4_, partially thawed state (*T* > *T*_m_*'*).

Drip loss was determined with a total of n = 9 (three replicates, each with three different measurements).

Level of significance: ^⁎⁎⁎^*p* < 0.001.

### 3.3 pH, TA, and RSC

The changes in pH, TA, and RSC of KWK samples during 4 weeks of frozen storage with or without phase transitions are shown in Table 3. The pH and TA are vital markers of kimchi maturity and quality (Kang et al., 2019). The initial pH and TA of KWK were 6.12 and 0.14 %, respectively. Over the 4 weeks of frozen storage, the pH and TA values of KWK samples ranged from 6.05–6.18 and 11–0.15 %, respectively, with no significant differences observed regardless of the phase transition (*p* > 0.05). The RSC of all frozen KWK samples remained consistent, ranging from 30.88 to 32.87 mg/g throughout the entire storage period, with no significant differences observed regardless of the phase transition (*p* > 0.05). These results indicate that the interaction effect that the storage period and temperature fluctuations have on these parameters was not significant (*p* > 0.05). Reducing sugars in kimchi, such as glucose, fructose, and maltose, serve as substrates for LAB and typically decrease because of fermentation during refrigerated storage (Park et al., 2023). The stable RSC values measured indicate that frozen storage inhibits LAB growth, thereby delaying fermentation, maintaining pH and TA levels, and preventing the negative flavours and odours associated with over-ripening (Kang et al., 2024; Park, Kim, Park, et al., 2024). Although *T*_4_ represents a partially thawed state, microbial activity may have been suppressed as a result of the low temperature itself and the fact that samples were stored at −45 °C for most of the storage period. It has been reported that microbial growth is generally halted below −18 °C, and metabolic activity is reduced, which positively contributes to the quality stability of food products (Alsailawi et al., 2020). Therefore, frozen storage can effectively maintain the physicochemical stability of kimchi by inhibiting fermentation and over-ripening, regardless of phase transition state or storage duration.

**Table 3:** Changes in the pH, titratable acidity, and reducing sugar content of frozen Korean white kimchi during 4 weeks of storage with or without phase transitions.

Physicochemical quality parameter	Temperature fluctuation	Storage period (week)					Levels of significance		
		0	1	2	3	4	SP	TF	SP × TF
pH	*T*_1_	6.12 ± 0.02^Aa^	6.14 ± 0.02^Aa^	6.14 ± 0.02^Aa^	6.15 ± 0.03^Aa^	6.10 ± 0.02^Aa^	*	NS	NS
	*T*_2_	6.12 ± 0.02^Aa^	6.18 ± 0.05^Aa^	6.13 ± 0.01^Aa^	6.09 ± 0.06^Aa^	6.05 ± 0.10^Aa^			
	*T*_3_	6.12 ± 0.02^Aa^	6.15 ± 0.01^Aa^	6.09 ± 0.02^Ba^	6.10 ± 0.04^Aa^	6.10 ± 0.09^Aa^			
	*T*_4_	6.12 ± 0.02^Aa^	6.14 ± 0.04^Aa^	6.09 ± 0.01^Ba^	6.07 ± 0.09^Aa^	6.06 ± 0.10^Aa^			
TA (%)	*T*_1_	0.14 ± 0.01^Aa^	0.13 ± 0.02^Aa^	0.12 ± 0.01^Aa^	0.15 ± 0.02^Aa^	0.14 ± 0.02^Aa^	NS	NS	NS
	*T*_2_	0.14 ± 0.01^Aa^	0.13 ± 0.02^Aa^	0.13 ± 0.01^Aa^	0.13 ± 0.02^Aa^	0.14 ± 0.03^Aa^			
	*T*_3_	0.14 ± 0.01^Aa^	0.15 ± 0.01^Aa^	0.13 ± 0.01^Aab^	0.11 ± 0.01^Ab^	0.13 ± 0.01^Aab^			
	*T*_4_	0.14 ± 0.01^Aa^	0.14 ± 0.01^Aa^	0.14 ± 0.01^Aa^	0.14 ± 0.02^Aa^	0.14 ± 0.02^Aa^			
RSC (mg/g)	*T*_1_	31.28 ± 1.07^Aa^	32.34 ± 2.84^Aa^	33.47 ± 2.08^Aa^	30.78 ± 2.70^Aa^	31.59 ± 3.07^Aa^	NS	NS	NS
	*T*_2_	31.28 ± 1.07^Aa^	31.91 ± 2.91^Aa^	33.87 ± 2.31^Aa^	30.91 ± 2.61^Aa^	33.01 ± 2.84^Aa^			
	*T*_3_	31.28 ± 1.07^Aa^	31.99 ± 2.86^Aa^	31.91 ± 2.56^Aa^	30.84 ± 2.66^Aa^	30.88 ± 1.33^Aa^			
	*T*_4_	31.28 ± 1.07^Aa^	32.21 ± 2.43^Aa^	33.87 ± 2.61^Aa^	31.24 ± 2.29^Aa^	33.01 ± 2.84^Aa^			

SP, storage period; TF, temperature fluctuation.

All data are presented as the mean ± standard deviation. Different uppercase letters (A–B) within the same column and different lowercase letters (a–b) within the same row indicate significant differences (*p* < 0.05). TA, titratable acidity; RSC, reducing sugar content; *T*_1_, glassy state without fluctuations (−45 °C); *T*_2_, glassy state (*T* < *T*_g_*'*); *T*_3_, rubbery state (*T*_g_*'* < *T* < *T*_m_*'*); *T*_4_, partially thawed state (*T* > *T*_m_*'*).

The pH, TA, and RSC were determined with a total of n = 9 (three replicates, each with three different measurements).

Level of significance: NS, not significant (*p* > 0.05); ^⁎^*p* < 0.05.

### 3.4 Instrumental hardness and colour

Instrumental hardness is a crucial metric for accurately assessing the texture and quality of cabbage kimchi (Kim et al., 2020). Changes in the instrumental hardness of KWK samples during 4 weeks of storage with or without phase transitions are shown in Table 4. The hardness of KWK immediately after freezing was 7.09 N. Importantly, hardness increased with the storage period and was influenced by phase transitions. After 4 weeks of frozen storage, hardness in the glassy state (*T*_1_ and *T*_2_) ranged from 7.98 to 8.70 N, which was significantly lower than the 9.26–9.96 N observed in *T*_3_ and *T*_4_ (*p* < 0.05). Two-way ANOVA revealed that the storage period, temperature fluctuations, and their interaction significantly affected KWK hardness (*p* < 0.001). The increase in hardness is primarily attributed to microstructural damage caused by the growth and distribution of ice crystals during storage. In *T*_1_ and *T*_2_, storage in the glassy state led to the formation of small, uniform ice crystals, resulting in minimal structural damage. In contrast, *T*_3_ and *T*_4_ experienced temperature fluctuations and entered rubbery or partially thawed states, where larger and more irregular ice crystals were formed, leading to increased drip loss and cell shrinkage (Kumar et al., 2020). Additionally, freeze-concentration and dehydration are believed to have enhanced tissue rigidity, further contributing to the increase in hardness. Our findings are consistent with previous research indicating that frozen cabbage kimchi stored in the rubbery state at −20 °C for 8 weeks undergoes ice crystal growth with increased drip loss, leading to cell wall shrinkage and increased fibre content in the cross-sectional area, ultimately increasing hardness after thawing (Kang et al., 2024). Additionally, studies indicate that storing plant-based foods, such as celery and potatoes, below their *T*_g_*'* mitigates hardness changes, indicating that glass-state storage is effective for salted vegetables (Kumar et al., 2019; Xu et al., 2020).

**Table 4:** Changes in the instrumental hardness of frozen Korean white kimchi during 4 weeks of storage with or without phase transitions.

Parameter	Temperature fluctuation	Storage period (week)					Levels of significance		
		0	1	2	3	4	SP	TF	SP × TF
Hardness (N)	*T*_1_	7.09 ± 0.81^Aa^	7.25 ± 0.92^Aa^	7.38 ± 0.78^Aa^	7.45 ± 0.87^Ba^	7.98 ± 1.10^Ba^	***	***	***
	*T*_2_	7.09 ± 0.81^Aa^	7.39 ± 0.46^Aa^	7.84 ± 1.60^Aa^	8.59 ± 1.08^ABa^	8.70 ± 0.54^ABa^			
	*T*_3_	7.09 ± 0.81^Ab^	7.45 ± 0.60^Ab^	7.80 ± 1.00^Aab^	8.12 ± 0.66^ABab^	9.26 ± 0.80^ABa^			
	*T*_4_	7.09 ± 0.81^Ac^	7.70 ± 0.52^Ac^	8.18 ± 1.00^Abc^	9.38 ± 0.91^Aab^	9.96 ± 0.90^Aa^			

SP, storage period; TF, temperature fluctuation.

All data are presented as the mean ± standard deviation. Different uppercase letters (A–B) within the same column and different lowercase letters (a–b) within the same row indicate significant differences (*p* < 0.05). *T*_1_, glassy state without fluctuations (−45 °C); *T*_2_, glassy state (*T* < *T*_g_*'*); *T*_3_, rubbery state (*T*_g_*'* < *T* < *T*_m_*'*); *T*_4_, partially thawed state (*T* > *T*_m_*'*).

Hardness was determined with a total of n = 9 (three replicates, each with three different measurements).

Level of significance: ^⁎⁎⁎^*p* < 0.001.

Colour is an easily detectable characteristic that influences consumer preferences and directly impacts sales (Yang et al., 2024). Changes in the CIE colour parameters (*L*^⁎^, *a*^⁎^, and *b*^⁎^) of frozen KWK samples during 4 weeks of storage with or without phase transitions are shown in Table 5. The initial CIE *L*^⁎^, *a*^⁎^, and *b*^⁎^ values after freezing were 65.58, −0.85, and 3.87, respectively. KWK stored under *T*_1_ and *T*_2_ conditions had *L*^⁎^ values between 65.38 and 68.93 over 4 weeks, whereas values exceeded 70 by the fourth and second weeks under *T*_3_ and *T*_4_ conditions, respectively. The *a*^⁎^ and *b*^⁎^ values of KWK stored for 4 weeks under stable temperature conditions (*T*_1_) were −1.33 and 4.30, respectively, which were significantly different from those of the group stored under fluctuating conditions (*p* < 0.05). Ice crystal growth during freezing leads to the destruction of cell walls and parenchymal tissues in plant matrices, resulting in structural damage (Kim et al., 2022). Such structural damage may alter the optical properties of the tissue after thawing, causing not only an increase in the *L*^⁎^ value but also changes in the *a*^⁎^ and *b*^⁎^ values as a result of the degradation or leakage of pigments. Notably, in the first week of storage, the Δ*E*^⁎^ value ranged from 1.55 to 1.94 across all groups, whereas by the fourth week, that of *T*_1_ was 1.79, and that of *T*_4_ significantly increased to 6.89 (*p* < 0.05). The ΔE* values of frozen KWK samples increased significantly with the storage period and were influenced by temperature fluctuations (*p* < 0.001). Consistent with our findings, previous studies reported less discolouration in celery frozen under glassy conditions compared with that under rubbery conditions with temperature fluctuations (Xu et al., 2020). These results suggest that temperature fluctuations causing phase transitions lead to discolouration and structural damage in frozen KWK, whereas stable storage in the glassy state mitigates these effects.

**Table 5:** Changes in the CIE colour values of frozen Korean white kimchi during 4 weeks of storage with or without phase transitions.

Colour parameter	Temperature fluctuation	Storage period (week)					Levels of significance		
		0	1	2	3	4	SP	TF	SP× TF
*L*^⁎^	*T*_1_	65.58 ± 2.40^Aa^	65.38 ± 1.67^Aa^	67.25 ± 0.36^Ba^	65.13 ± 0.93^Ba^	65.93 ± 1.76^Ba^	***	***	***
	*T*_2_	65.58 ± 2.40^Aa^	66.82 ± 0.94^Aa^	67.17 ± 0.69^Ba^	67.19 ± 2.66^Ba^	68.93 ± 2.32^ABa^			
	*T*_3_	65.58 ± 2.40^Ab^	66.48 ± 1.41^Ab^	67.19 ± 2.66^Bab^	68.42 ± 2.72^ABab^	70.63 ± 0.75^Aa^			
	*T*_4_	65.58 ± 2.40^Ac^	67.93 ± 2.41^Abc^	70.65 ± 1.00^Aab^	71.63 ± 1.51^Aa^	72.03 ± 2.05^Aa^			
*a*^⁎^	*T*_1_	−0.85 ± 0.09^Aa^	−0.88 ± 0.15^Aab^	−1.04 ± 0.21^Aab^	−1.15 ± 0.15^Abc^	−1.33 ± 0.09^Ac^	***	***	***
	*T*_2_	−0.85 ± 0.09^Aa^	−1.05 ± 0.15^Aab^	−1.13 ± 0.25^Aab^	−1.19 ± 0.14^ABb^	−1.51 ± 0.11^Ac^			
	*T*_3_	−0.85 ± 0.09^Aa^	−1.10 ± 0.30^Aab^	−1.31 ± 0.26^ABb^	−1.48 ± 0.25^BCbc^	−1.79 ± 0.12^Bc^			
	*T*_4_	−0.85 ± 0.09^Aa^	−1.01 ± 0.11^Aa^	−1.55 ± 0.12^Bb^	−1.75 ± 0.11^Cbc^	− 1.83 ± 0.11^Bc^			
*b*^⁎^	*T*_1_	3.87 ± 0.42^Aa^	4.58 ± 0.66^Aa^	4.56 ± 1.01^Ca^	4.41 ± 0.43^Ca^	4.30 ± 0.28^Ca^	***	***	***
	*T*_2_	3.87 ± 0.42^Ad^	4.42 ± 0.75^Acd^	5.45 ± 0.46^BCbc^	5.97 ± 0.78^Bab^	6.91 ± 0.30^Ba^			
	*T*_3_	3.87 ± 0.42^Ab^	4.93 ± 0.93^Ab^	6.26 ± 0.84^ABa^	6.86 ± 0.52^ABa^	7.29 ± 0.51^Ba^			
	*T*_4_	3.87 ± 0.42^Ab^	4.54 ± 0.56^Ab^	7.30 ± 1.01^Aa^	7.59 ± 0.74^Aa^	8.39 ± 0.36^Aa^			
Δ*E*^⁎^	*T*_1_	–	1.66 ± 0.59^Aa^	1.93 ± 0.44^Aa^	1.17 ± 0.46^Ca^	1.79 ± 0.23^Ca^	***	***	***
	*T*_2_	–	1.55 ± 0.82^Ab^	2.08 ± 0.61^Aab^	3.02 ± 1.29^BCab^	4.13 ± 1.57^BCa^			
	*T*_3_	–	1.88 ± 0.55^Ab^	3.17 ± 1.04^Ab^	3.87 ± 1.76^Bab^	5.47 ± 0.73^ABa^			
	*T*_4_	–	1.94 ± 1.38^Ab^	3.53 ± 1.42^Ab^	6.44 ± 1.39^Aa^	6.89 ± 1.95^Aa^			

SP, storage period; TF, temperature fluctuation.

All data are presented as the mean ± standard deviation. Different uppercase letters (A–C) within the same column and different lowercase letters (a–d) within the same row indicate significant differences (*p* < 0.05). *T*_1_, glassy state without fluctuations (−45 °C); *T*_2_, glassy state (*T* < *T*_g_*'*); *T*_3_, rubbery state (*T*_g_*'* < *T* < *T*_m_*'*); *T*_4_, partially thawed state (*T* > *T*_m_*'*).

CIE colour was determined with a total of n = 9 (three replicates, each with three different measurements).

Level of significance: ^⁎⁎⁎^*p* < 0.001.

### 3.5 Antioxidant activity

**Fig. 3:**
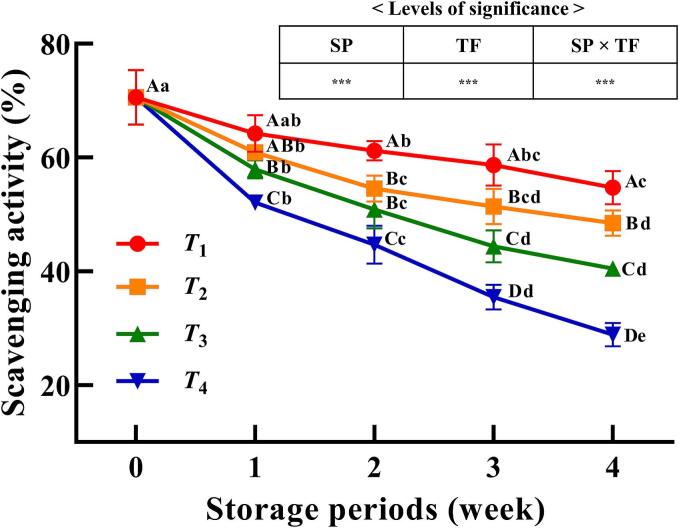
Antioxidant activities of frozen Korean white kimchi during four weeks of storage with or without phase transitions. SP, storage period; TF, temperature fluctuation. *T*_1_, glassy state without temperature fluctuations (−45 °C); *T*_2_, glassy state (*T* < *T*_g_*'*); *T*_3_, rubbery state (*T*_g_*'* < *T* < *T*_m_*'*); *T*_4_, partially thawed state (*T* > *T*_m_*'*). *T*_2_–*T*_4_ were stored under fluctuating temperature conditions. Antioxidant activities were determined with a total of *n* = 9 (three replicates, each with three different measurements). Level of significance: ^⁎⁎⁎^*p* < 0.001.

The antioxidant effects of kimchi, driven by phenolic compounds, vitamin C, and phenolic acids, are significantly influenced by storage conditions (Park et al., 2011; Park, Kim, Park, et al., 2024). Changes in the antioxidant activities of KWK samples during 4 weeks of storage with or without phase transitions are shown in Fig. 3. The antioxidant activity of KWK immediately after freezing was 70.60 % and had been affected by temperature fluctuations and storage duration. After 4 weeks of storage, frozen KWK samples in the glassy state (*T*_1_ and *T*_2_) maintained significantly higher antioxidant activity (48.45–54.70 %) compared with those in the partially freeze-concentrated (*T*_3_; 40.46 %) and rubbery (*T*_4_; 28.86 %) states (*p* < 0.05). The antioxidant activity of frozen KWK samples decreased significantly with the storage period and was affected by temperature fluctuations and their interaction (*p* < 0.001). These results are consistent with previous studies showing that frozen storage conditions significantly impact the preservation of antioxidant properties in plant foods (Neri et al., 2020). A decrease in antioxidant activity and phenolic content with increasing storage duration has been previously reported in cabbage kimchi frozen at −18 °C (Park, Kim, Park, et al., 2024).

In frozen mangoes, the content of vitamin C, which has potent antioxidant activity, decreases during frozen storage with temperature fluctuations, whereas glassy-state storage mitigates this reduction (Zhang et al., 2018). Similarly, a previous study demonstrated that during 20 days of frozen storage at −25 °C, the antioxidant activity of pomegranate juice decreased as a result of the oxidation of anthocyanins and phenolic compounds, reducing its DPPH radical-scavenging properties (Mirsaeedghazi et al., 2014). Moreover, cell damage caused by freezing can promote the release of antioxidant compounds and accelerate chemical and enzymatic oxidation reactions, thereby reducing antioxidant activity compared to fresh products (Neri et al., 2020). Therefore, frozen storage in the glassy state without temperature fluctuations is effective in minimising structural cell damage and preserving the antioxidant activity of KWK.

### 3.6 PCA

**Fig. 4:**
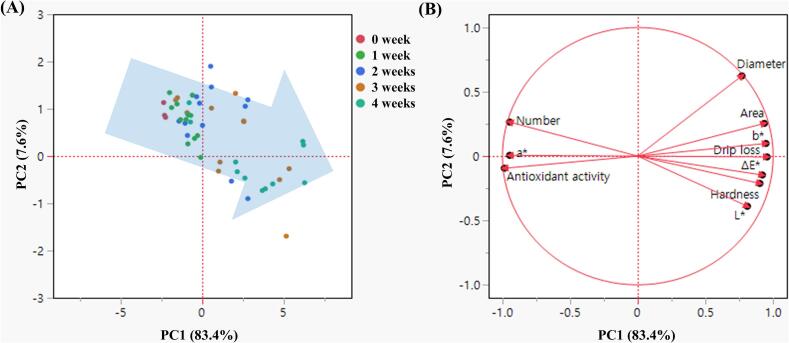
Principal component analysis of physicochemical quality attributes of frozen Korean white kimchi during 4 weeks of storage. (A) Score plot showing sample distribution by storage week; the directional arrow indicates the gradual shift in overall quality. (B) Loading plot illustrating the contributions of quality variables to PC1 and PC2. *T*_1_, glassy state without temperature fluctuations (−45 °C); *T*_2_, glassy state (*T* < *T*_g_*'*); *T*_3_, rubbery state (*T*_g_*'* < *T* < *T*_m_*'*); *T*_4_, partially thawed state (*T* > *T*_m_*'*). *T*_2_–*T*_4_ were stored under fluctuating temperature conditions.

**Fig. 5:**
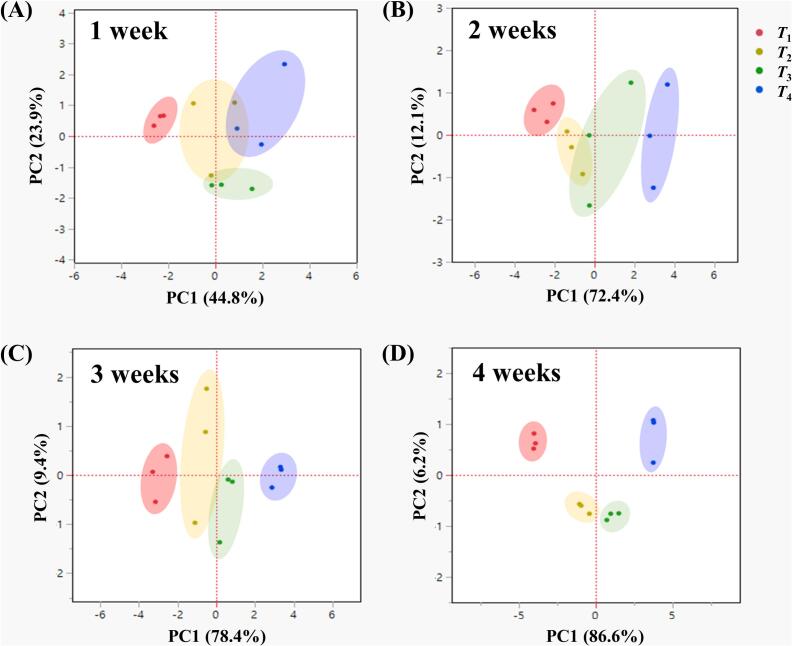
Score plots of frozen Korean white kimchi samples stored for (A) 1 week, (B) 2 weeks, (C) 3 weeks, and (D) 4 weeks. *T*_1_, glassy state without temperature fluctuations (−45 °C); *T*_2_, glassy state (*T* < *T*_g_*'*); *T*_3_, rubbery state (*T*_g_*'* < *T* < *T*_m_*'*); *T*_4_, partially thawed state (*T* > *T*_m_*'*). *T*_2_–*T*_4_ were stored under fluctuating temperature conditions.

To comprehensively evaluate the multivariate quality variation of frozen KWK under different phase transition conditions, PCA was performed using 10 standardised variables that showed significant differences depending on the storage period and phase transition state. The analysed variables included ice crystal parameters (number, diameter, and area), drip loss, hardness, colour values (*L*^⁎^, *a*^⁎^, *b*^⁎^, and Δ*E*^⁎^), and antioxidant activity. Fig. 4 presents the overall PCA results based on storage period. The first and second principal components (PC1 and PC2) accounted for 83.4 % and 7.6 % of the total variance, respectively, with a cumulative explained variance of 91.0 % (Fig. 4A). As storage time increased, most samples shifted toward the positive direction of PC1, indicating a gradual change or deterioration in quality attributes. The loading plot (Fig. 4B) showed that drip loss, ice crystal diameter and area, hardness, and colour values (*L*^⁎^, *b*^⁎^, and Δ*E*^⁎^) were positively associated with PC1, suggesting that increases in these variables contributed to quality degradation. In contrast, antioxidant activity and the number of ice crystals were negatively associated with PC1, indicating that their reduction was linked to quality decline. The score plots in Fig. 5 illustrate sample distributions by storage week according to phase transition state (week 0 samples were excluded from the analysis because all groups shared the same samples immediately after freezing). Some group overlap was observed at week 1; however, as storage progressed, samples from temperature-fluctuated conditions (*T*_2_, *T*_3_, and *T*_4_) gradually shifted toward the positive PC1 axis. By week 4, the groups were clearly separated, indicating that phase transition states caused by temperature fluctuations during storage had a significant impact on quality characteristics. These PCA results are consistent with previous studies reporting that temperature fluctuations promote ice recrystallisation and physical degradation (Syamaladevi et al., 2012; Vicent et al., 2020), whereas storage below *T*_g_*'* without temperature fluctuation effectively maintains antioxidant activity, structural integrity, and microbial stability (Kang et al., 2024; Kumar et al., 2019; Park, Kim, Lee, et al., 2024; Park, Kim, Park, et al., 2024; Zhang et al., 2018). Taken together, storing KWK in the glassy state under stable temperatures appears to be the most effective strategy for maintaining its quality during long-term frozen storage.

## 4 Conclusion

This study evaluated the effects of phase transitions during 4 weeks of frozen storage on the physicochemical properties of KWK, including ice crystal formation, microstructure, drip loss, hardness, colour, and antioxidant activity. Although pH, TA, and RSC remained stable, significant differences were observed in other quality parameters depending on the phase transition state. Notably, samples stored in the glassy state below *T*_g_*'* without temperature fluctuations exhibited suppressed ice crystal growth and structural damage, along with better retention of drip loss, hardness, colour, and antioxidant activity. PCA further revealed that, as storage progressed, quality attributes became increasingly distinct based on the phase transition states induced by temperature fluctuations. These findings suggest that maintaining KWK in the glassy state under stable frozen conditions is effective in preserving its quality during long-term storage. However, this study primarily focused on physicochemical assessments under controlled phase transition conditions. Microbial analysis, sensory evaluation, and the effects of different thawing methods were not included, and the storage duration was limited to 4 weeks. Future research should explore these additional factors by conducting comprehensive evaluations of physicochemical, sensory, and microbial properties under extended frozen storage and diverse thawing conditions.

## CRediT authorship contribution statement

**Miran Kang:** Writing – review & editing, Writing – original draft, Visualization, Validation, Formal analysis, Data curation, Conceptualization. **Eunji Kim:** Validation, Methodology, Formal analysis, Data curation. **Hyun-Jung Chung:** Writing – review & editing, Validation, Supervision, Resources, Methodology, Conceptualization. **Sung Hee Park:** Writing – review & editing, Validation, Supervision, Software, Resources, Project administration, Methodology, Funding acquisition.

## Declaration of competing interest

The authors declare that they have no known competing financial interests or personal relationships that could have appeared to influence the work reported in this paper.

## Data Availability

Data will be made available on request.
